# The Antibacterial Efficacy of Photo-Activated Disinfection, Chlorhexidine and Sodium Hypochlorite in Infected Root Canals: An *in Vitro* Study

**DOI:** 10.7508/iej.2016.03.006

**Published:** 2016-05-01

**Authors:** Mohammad Samiei, Shahriar Shahi, Amir Ardalan Abdollahi, Mahsa Eskandarinezhad, Ramin Negahdari, Zahra Pakseresht

**Affiliations:** a *Department of Endodontics, Dental and Periodontal Research Center, Dental School, Tabriz University of Medical Sciences, Tabriz, Iran; *; b * Department of Prosthodontics, Dental and Periodontal Research Center, Dental School, Tabriz University of Medical Sciences, Tabriz, Iran; *; c *Private Practice, Urmia, Iran*

**Keywords:** *Enterococcus faecalis*, Laser, Photo-Activated Disinfection, Photodynamic Therapy, Sodium Hypochlorite

## Abstract

**Introduction:**

This study compared the efficacy of light-activated low-power laser, 2% chlorhexidine (CHX) and 2.5% NaOCl in eliminating *Enterococcus faecalis* (*E. faecalis)* from the root canal system.

**Methods and Materials:**

The root canals of 60 maxillary central incisors were contaminated with *E. faecalis* and then the bacteria were incubated for 24 h. All the root canals were instrumented in a crown-down manner with #4 and 3 Gates-Glidden drills, followed by RaCe rotary files (40/0.10, 35/0.08, and 30/0.06). The samples were randomly assigned to three experimental groups and one control group (*n*=15). In the control group no intervention was made. In the photo-activated disinfection (PAD) group, laser therapy was undertaken with diode laser beams (with an output power of 100 mW/cm^2^) for 120 sec. For the other two experimental groups, root canals were irrigated either with 5 mL of 2% CHX or 2.5% NaOCl solutions, respectively. The Kruskal-Wallis test was used to compare the CFU values of the bacteria and post-hoc Bonferroni test was used for pairwise comparisons. The level of significance was set at 0.05.

**Results:**

The inhibition of bacterial growth in all the experimental groups was significantly superior to the control group (*P*<0.05). There was no significant difference between the effect of PAD and 2% CHX (*P*=0.05). The effect of 2.5% NaOCl was significantly better than that of the PAD technique (*P*<0.001). In addition, 2.5% NaOCl was significantly better than 2% CHX (*P*=0.007).

**Conclusion:**

Photodynamic therapy was effective in reducing the *E. faecalis* counts in comparison with the control group, but 2.5% NaOCl solution was the most effective protocol.

## Introduction

The main aim of root canal treatment is to achieve a root canal system free from damaging irritants; because the residual microorganisms in necrotic pulps might cause persistent inflammation in periradicular tissues and treatment failure [1, 2]. Many microorganisms such as* Enterococcus faecalis* (*E. faecalis*) play important roles in the etiology of persistent periradicular lesions after root canal treatment [1, 3]. *E. faecalis* has been found in 24-77% of the cases of teeth with treatment resistant periradicular lesions [3, 4]. 

It is well established that complete debridement and thorough elimination of bacteria from the root canal is very difficult, if not impossible, because of the complexity of the root canal system [5-7]. Thus in addition to mechanical preparation, it is highly recommended to use disinfecting irrigants, due to their ability to dissolve organic and inorganic tissues, lubricate the root canal and eliminate bacteria and their by-products [8, 9]. 

Sodium hypochlorite (NaOCl) is an irrigation solution predominantly used in endodontic treatment in concentrations ranging from 0.5 to 5.25%, although alternative solutions have already been studied [10]. The use of chlorhexidine gluconate (CHX) as an irrigant during root canal therapy has been suggested based on its antibacterial effect, substantivity and milder malodor and cytotoxicity in comparison with NaOCl [11]. In spite of the disinfecting effect of CHX, it is unable to eliminate necrotic tissues from the root canals and remove the smear layer. In addition, it may cause toxicity, induce an inflammatory response and in some cases result in allergic reactions [12, 13]. 

Researchers have studied alternative techniques due to the presence of a smear layer that reduces the efficacy of disinfectants [14] and the complexity of the root canal system which makes it impossible to completely eliminate debris and achieve a sterile root canal by the use of irrigating solutions [15].

Photo-activated disinfection (PAD) (*aka *photodynamic therapy, PDT) is a novel method of disinfection for use in both caries removal and root canal treatment [16]. The laser light is thought to be able to reach areas that are inaccessible with conventional techniques [17]. High-power lasers such as Nd: YAG and Er: YAG may induce periradicular necrosis and charring of dentinal tubules through generation of heat. The new method for eradication of microorganisms from the root canal is the application of low-power lasers [18, 19]. PAD is an antimicrobial strategy in which low-energy laser is used to activate a nontoxic photosensitizer like tolonium chloride, and the singlet oxygen released from these dyes damages the membranes and DNA of microorganisms [19, 20]. It has been recommended for use in root canal treatment as an alternative or supplement to other disinfection methods [21-23] since it produces heat that is not clinically significant (less than 0.5^°^C) [19]. In addition, photosensitizers have a high degree of selectivity to kill microorganisms without affecting the host cell viability. Application of PAD has shown to be successful in the eradication of multi-drug-resistant microorganisms [24]. According to a study by Fonseca *et al. *[25], this method is very effective in eliminating *E. faecalis* from the root canal system.

The aim of this *in vitro* study was to compare the antibacterial activities of photo-activated low-level lasers and two conventional irrigation methods naming 2% CHX and 2.5% NaOCl against *E. faecalis* in infected root canals.

## Materials and Methods

Approval of this project was obtained from the Research and Ethics Committee of Tabriz University of Medical Sciences, Tabriz, Iran (Grant No.:1357) Sixty extracted human maxillary central incisors were selected for this study. All the teeth were extracted because of periodontal disease, and had completely developed single roots without caries, previous endodontic treatment and anomalies. Following extraction, each tooth was stored in 3% chloramine-T solution at 4^°^C. The external root surface was cleaned with ultrasonic tips to remove the remnants of periodontal soft tissues. Teeth were selected with apical foramina approximately matching the size of a #25 K-Flexofile (Dentsply, Maillefer, Ballaigues, Switzerland). Also teeth with cracks and calcifications in radiographic views were excluded. The teeth were decoronated to a standard 12-mm root segment. The working length was determined with #25 K-Flexofile (Dentsply, Maillefer, Ballaigues, Switzerland), 1 mm short of the apical foramen. All the root canals were instrumented in a crown-down manner. The coronal two-thirds of the canals were prepared with #4 and 3 Gates-Glidden drills (Dentsply, Maillefer, Ballaigues, Switzerland), followed by the use of 40/0.10, 35/0.08 and 30/0.06 RaCe rotary instruments (FKG Dentaire, La Chaux-de-Fonds, Switzerland). The size of master apical file was established at #40. Each canal was irrigated with 1 mL of normal saline solution throughout the instrumentation sequence. The smear layer was removed using 1 mL of 17% ethylenediaminetetraacetic acid (EDTA) (Pulpdent Corp., Watertown, MA, USA) for 3 min, followed by a final rinse with 1 mL of 5.25% NaOCl (Taj Corp, Tehran, IRI) for 3 min. The teeth were sterilized by autoclaving at 121^°^C and 15 psi pressure for 20 min. To confirm sterilization, the teeth were incubated in brain-heart infusion (BHI) broth (Merck, Darmstadt, Germany) at 37^°^C for 24 h.

A purified culture of *E. faecalis* (ATCC 29212, Reference Laboratories of Iran Research Center, Tehran, Iran) was provided. Then bacteria were incubated in BHI broth at 37^°^C for 24 h under aerobic conditions. The grown colonies were used to inoculate blood agar broth (Merck, Darmstadt, Germany) and incubated at 37^°^C for 24 h. A spectrophotometer was used to determine the *E. faecalis* culture in blood agar broth as 2.5×10^8^ colony forming units in mL (CFU/mL). Then 200 µL of the bacterial culture were transferred into the canal lumen using a micropipette. After 48 h, all the root canals were dried with sterile paper points [26].

***Experimental groups***

The 60 samples were randomly divided into three experimental groups and one control group (*n*=15). The experimental groups were subjected to each of the following experimental treatment protocols: group 1, PAD; after placement of 1.2 mg/mL of tolonium chloride for 30 sec, the root canals were irradiated with a diode laser beam (B&W TEK Inc., Newark, DE, USA) with a power output of 100 mW/cm^2^ and 635 nm of wavelength for 120 sec, using a flexible Endo tip (Denfotex Technologies Ltd., Inverkeithing, Fife, UK) measuring 15 mm in length and 300 µm in diameter (4), group 2,CHX; the root canals were irrigated with 5 mL of 2% CHX (Perio-Kin, Laboratories Kin, Barcelona, Spain) for 60 sec, and group 3, NaOCl; the root canals were irrigated with 5 mL of 2.5% NaOCl for 60 sec. In the control group no other procedures were carried out. Then all the teeth (control and experimental groups) were placed in a freezer at -25^°^C to prevent *E. faecalis* from being killed by the heat produced during drilling for sampling procedures [26, 27]. 

The efficacy of disinfection was evaluated by collecting 10 µg of dentin shavings from each canal by drilling the walls of canals using #5 and 6 Gates-Glidden drills. The drills were inserted into the canals until they reached 1 mm short of the working length. The samples were transferred into tubes containing 2 mL of normal saline and vortexed for 20 sec. Serial dilutions of 10 times were provided up to 10^-7^. Then 100 µL of each solution was added to three plates of agar blood culture and incubated at 37^°^C for 48 h. All the procedures were carried out in a laminar flow chamber with sterile instruments and by obtaining aseptic conditions. A classic colony counting technique was used for counting the *E. faecalis* bacteria in blood agar plates. The average CFU values of plates related to concentrations of 10^-2^, 10^-3^ and 10^-4^ were counted. For the clarity and deletion of less significant measurements, the bacterial growth in agar plates related to the concentrations of 10^-5^, 10^-6^ and 10^-7^ was not considered. 

***Statistical analysis***

Statistical analysis was performed using SPSS software (SPSS version 20.0, SPSS, Chicago, IL, USA). The Kolmogorov-Smirnov test showed that the data of the study was non-parametric. Therefore the Kruskal-Wallis test was used to compare the CFU values of the bacteria and post-hoc Bonferroni test was used for pairwise comparisons. The level of significance was set at 0.05. 

## Results

Table 1 presents the bacterial counts in four groups. The results of Kruskal-Wallis test showed statistically significant differences between the groups (*P*<0.05). The inhibition of bacterial growth in all the experimental groups was significantly superior to the control group. The results of pairwise analysis using post hoc Bonferroni test are presented in Table 2. 

The effect of PAD and 2% CHX solution were significantly different at 10^-2^ concentration (*P*<0.05). The effect of NaOCl at all concentrations was significantly better than PAD (*P*<0.05).

The effect of NaOCl at mean dilutions was superior to that of 2% CHX (*P*=0.007). In comparison to the control group, the residual bacteria in PAD, NaOCl and CHX groups was 4.56, 2.93 and 0.82%, respectively.

## Discussion

In this study, we compared the antibacterial effects of photo-activated low-level lasers, 2% CHX and 2.5% NaOCl on *E. faecalis* in infected root canals. The results of this study showed that all the three antibacterial agents significantly decreased CFU counts of *E. faecalis* compared to the control group. However, there was no significant difference between PAD and 2% CHX solution; also the efficacy of 2.5% NaOCl was superior to the other antibacterial agents.

*E. faecalis* is able to produce extra- and intra-radicular biofilms which are very difficult to eliminate from the infected root canals [28, 29]. On the other hand, many of the common antibacterial agents may have no effect on the deep layers of dentin [30]. Various types of lasers have been used in dentistry, particularly in endodontics, exhibiting some efficacy in eradication of *E. faecalis *[31-35]. The advantage of laser in this respect has been stated as having the ability to control and set the depth of light penetration, resulting in accessibility to areas with complex structures [36, 37].

The use of low-power lasers in PDT is harmless to human tissues and the temperature increase is very low [37-39]. Dickers *et al.* [38] reported that heat production was proportional to the duration of laser application. Thus during the use of high-power lasers, their fibers should have a circular movement and be placed out of the root canal. In addition, sufficient intervals should be applied between laser applications until the surrounding tissues are cooled. However, the risk of temperature increase for hard and hard and soft tissues in PDT using low-power lasers is minimal and water coolant spray is not required [38, 40]. The other advantage of application of PDT in canals with curvature and structures like delta is that the thin and elastic tip of the light source could penetrate up to the apical areas. According to research, use of PDT alone and along with other disinfection methods is effective in the eradication of bacteria in these areas up to 95% and 98%, respectively [41]. Photo-sensitive materials can penetrate into the dentinal tubules and may be effective in eliminating bacterial colonies [40].

**Table 1:** Mean (SEM) [standard error of mean IQR (Interquartile range)] of bacterial counts at different dilutions between the test materials

**Test groups (Dilutions)**	**2% Chlorhexidine**	**Photo-activated laser**	**5% NaOCl**	**Control**
**10** ^-2^	1.53 (0.27)	2.33 (0.19)	0.33 (0.19)	46.5 (1.35)
**10** ^-3^	1.13 (0.31)	1.8 (0.19)	0.47 (0.27)	38.33 (1.18)
**10** ^-4^	0.73 (0.23)	1.2 (0.21)	0.2 (0.2)	32.53 (1.0)
**Mean**	1.13 (0.25)	1.82 (0.15)	0.33 (0.19)	38.82 (0.88)

**Table 2:** Pairwise comparison of groups in terms of the bacterial growth in different dilutions. Reported data are *P*-values

**Groups**	**10** ^-2^	**10** ^-3^	**10** ^-4^	**Mean dilution**
**Laser-Chlorhexidine**	0.03	0.05	0.09	0.05
**NaOCl-Chlorhexidine**	0.002	0.05	0.03	0.007
**Laser-NaOCl**	<0.001	<0.001	<0.001	<0.001
**Chlorhexidine-Control**	<0.001	<0.001	<0.001	<0.001
**Laser-Control**	<0.001	<0.001	<0.001	<0.001
**NaOCl-Control**	<0.001	<0.001	<0.001	<0.001

In a systematic review, Arneiro *et al.* [34] concluded that PDT had better antimicrobial effects when used as an adjunct to NaOCl during endodontic treatment. In accordance with our study, Vaziri *et al.* [42] reported that PDT was less effective than 2.5% NaOCl in reducing *E. faecalis* counts and also combination of PDT and 2.5% NaOCl exhibited maximum efficacy. Furthermore, Meire *et al.* [43] reported that 2.5% NaOCl was very effective in elimination of *E. faecalis* biofilms from dentin disks and PDT resulted in an insignificant reduction in *E. faecalis* counts. However, Yildirim *et al.* [44] and Xhevdet *et al.* [32] reported that PDT was as effective as conventional 5% and 2.5% NaOCl irrigation regarding efficacy against *E. faecalis*, respectively. 

Rios *et al.* [22] demonstrated that a combination of PDT and irrigation with NaOCl was an efficient technique in decreasing bacterial load of the root canal system since the survival rate of *E. faecalis* in this group was 0.1%, whereas in root canals treated with PDT alone the survival rate was 2.9%. Recently, Komine and Tsujimoto [45] showed that 0.01‒0.001% methylene blue was effective in the application of PDT in root canals contaminated with *E. faecalis*.

In this study we found that the effect of NaOCl at mean dilutions was superior to that of 2% CHX. In contrast to this finding, Ahangari *et al. *[46] concluded that there was no difference between these solutions in terms of their antimicrobial effect on *E. faecalis*, which can be attributed to different methods. 

The results of the present study showed that the load of remaining bacteria in the group receiving photodynamic therapy decreased significantly compared to the control group. Although this reduction was lower than the conventional irrigation solutions to some extent, it demonstrated the significant role of this technique in eradication of one of the most resistant microorganisms from the root canal system. The best results of antibacterial efficacy were obtained with the use of NaOCl. However, to determine the most effective endodontic disinfection protocol, the efficacy of the techniques should be further determined with various bacterial species in root canals. Finally, it is necessary to evaluate the real contribution of PAD method to conventional chemomechanical preparation *in vivo*.

## Conclusion

Based on the results of this *in vitro* study, photodynamic therapy was as effective in reducing *Enterococcus faecalis* counts as chlorhexidine, but this effect was less than that of 2.5% sodium hypochlorite irrigation solution.
